# Genetic and epigenetic characteristics of human multiple hepatocellular carcinoma

**DOI:** 10.1186/1471-2407-10-530

**Published:** 2010-10-06

**Authors:** Kazuya Taniguchi, Terumasa Yamada, Yo Sasaki, Kikuya Kato

**Affiliations:** 1 Research Institute, Osaka Medical Center for Cancer and Cardiovascular Diseases, 1-3-3 Nakamichi, Higashinari-ku, Osaka, 537-8511, Japan; 2 Department of Surgery, Osaka Medical Center for Cancer and Cardiovascular Diseases, 1-3-3 Nakamichi, Higashinari-ku, Osaka, 537-8511, Japan

## Abstract

**Background:**

Multiple carcinogenesis is one of the major characteristics of human hepatocellular carcinoma (HCC). The history of multiple tumors, that is, whether they derive from a common precancerous or cancerous ancestor or individually from hepatocytes, is a major clinical issue. Multiple HCC is clinically classified as either intratumor metastasis (IM) or multicentric carcinogenesis (MC). Molecular markers that differentiate IM and MC are of interest to clinical practitioners because the clinical diagnoses of IM and MC often lead to different therapies.

**Methods:**

We analyzed 30 multiple tumors from 15 patients for somatic mutations of cancer-related genes, chromosomal aberrations, and promoter methylation of tumor suppressor genes using techniques such as high-resolution melting, array-comparative genomic hybridization (CGH), and quantitative methylation-specific PCR.

**Results:**

Somatic mutations were found in *TP53 *and *CTNNB1 *but not in *CDKN2A *or *KRAS*. Tumors from the same patient did not share the same mutations. Array-CGH analysis revealed variations in the number of chromosomal aberrations, and the detection of common aberrations in tumors from the same patient was found to depend on the total number of chromosomal aberrations. A promoter methylation analysis of genes revealed dense methylation in HCC but not in the adjacent non-tumor tissue. The correlation coefficients (*r*) of methylation patterns between tumors from the same patient were more similar than those between tumors from different patients. In total, 47% of tumor samples from the same patients had an *r *≥ 0.8, whereas, in contrast, only 18% of tumor samples from different patients had an *r *≥ 0.8 (p = 0.01). All IM cases were highly similar; that is, *r *≥ 0.8 (*p *= 0.025).

**Conclusions:**

The overall scarcity of common somatic mutations and chromosomal aberrations suggests that biological IM is likely to be rare. Tumors from the same patient had a methylation pattern that was more similar than those from different patients. As all clinical IM cases exhibited high similarity, the methylation pattern may be applicable to support the clinical diagnosis of IM and MC.

## Background

Human hepatocellular carcinoma (HCC) is one of the leading causes of death in Asian countries. Unlike cancers that are prevalent in other developed countries, HCC is characterized by underlying viral etiologic factors, such as hepatitis B virus (HBV) and hepatitis C virus (HCV). In Japan, HCV infection is the most common cause of HCC. One characteristic of HCC is a high rate of tumor recurrence [1-4], owing to multiple carcinogenesis. Multiple carcinogenesis is uncommon, except in HCC and some types of lung cancer. Multiple HCC is classified as either intrahepatic metastasis (IM) or multicentric carcinogenesis (MC) based on clinicopathological criteria [5,6]. Some groups have reported that IM recurrence develops earlier than MC, which leads to a poorer prognosis for IM than for MC [2,7]. Therefore, surgery may not be warranted for recurrent metastatic nodules, whereas, for MC lesions, radical surgery should be initially attempted if a functional liver reserve is adequate.

Numerous studies have investigated the genetic aberrations in HCC [8]. Somatic mutations in genes, such as *TP53*, have frequently been observed. Recurring allelic gains and losses on 14 chromosome arms have been detected in more than 30% of HCC cases [9-11]. These observations have been confirmed using array-comparative genomic hybridization (CGH) [12-14]. In addition to these genetic changes, epigenetic changes have also been extensively analyzed. Dense methylation of cancer-related genes is a characteristic of HCC [15]. Geographic variations in methylation status indicate that environmental factors affect the methylation status of genes in HCC [15]. In addition, the aberrant hypermethylation has been observed in non-neoplastic liver cells from patients with hereditary hemochromatosis [16].

As previously described, a history of multiple tumors in a single patient has been an important clinical issue. If there were multiple genomic aberrations, the lineage of multiple tumors could be deduced from the patterns of the aberrations. Genetic and epigenetic factors have been examined for this purpose. These factors include p53 mutation status [17], HBV integration sites [18], chromosome aberration [19-22], and methylation status [23]; however, despite these reports, no consensus leading to clinical application has been established. This is not surprising because the biological and genetic bases of IM and MC remain obscure.

In this study, we analyzed 30 multiple tumors from 15 patients for somatic mutations of cancer-related genes or chromosomal aberrations (i.e., allelic gains and losses) and the promoter methylation status of cancer-related genes using the latest techniques, such as high-resolution melting [24], array-CGH [25], and quantitative methylation-specific PCR (QMSP) [26]. We examined whether multiple HCC has specific molecular changes that indicate the process of carcinogenesis. We also evaluated whether these changes could be applicable to the differentiation of IM and MC.

## Methods

### HCC samples

A total of 30 tumor tissues and adjacent non-tumor tissues were obtained from 15 HCC patients who underwent their first surgical operation between 1998 and 2006. Tissues were stored at -80°C until further use. DNA was extracted from the frozen tumor tissues and adjacent non-tumor tissues using a QIAamp DNA Micro kit (Qiagen, Valencia, CA). This study conformed to the ethical guidelines of the 1975 Declaration of Helsinki and was approved by the ethical committee of Osaka Medical Center for Cancer and Cardiovascular Diseases. Informed consent was obtained from all of the investigated patients.

### Mutation analysis

Mutation screening was performed using high-resolution melting on a LightScanner (Idaho Technology Inc., Salt Lake City, UT) [24] according to the manufacturer's protocol, which was then followed by direct sequencing of the PCR products. The primer sequences that were used for both assays are provided in Additional file 1, Table S1.

### Analysis of chromosomal aberration

Array-CGH was performed using a 44K array (Agilent Technologies, Santa Clara, CA) according to the manufacturer's protocol [25]. Gains and losses that spanned fewer than 100 probes were omitted from the results in order to make the output comparable to those from previous studies that were carried out using conventional CGH or microsatellite markers. The extraction of data from images was carried out using the Feature Extraction Software (Agilent Technologies), and gains and losses were identified using the DNA Analysis Software (Agilent Technologies): the ADM-2 algorithm was employed using 8.0 as the threshold and 0.3219 as the minimum absolute average log ratio for the region. The array-CGH data was submitted to NCBI GEO (accession number, GSE22635).

### Methylation analysis

Genomic DNA was subjected to bisulfite treatment before methylation analysis, as previously described [27]. QMSP was performed as previously described [26] using TaqMan technology. The primer and probe sequences that were used are given in Additional file 1, Table S1. In order to prepare the positive control (i.e., 100% methylated DNA), we treated a mixture of genomic DNA from five lung cancer tissues with Sss I (CpG) methylase (New England Biolabs, Inc., Beverly, MA). In order to convert the nonmethylated cytosine residues into uracil, genomic DNA was treated with sodium bisulfite using the MethylEasy DNA Bisulphite Modification kit (TAKARA, Kyoto, Japan). Whereas a 5-methyl cytosine within the CpG islands remained unaltered, 4 μg of DNA was denatured by NaOH and modified by sodium bisulfite at 55°C for 12 hr. The DNA samples were then purified by isopropanol precipitation, washed with 70% ethanol, and resuspended in 50 μl water. The samples were then incubated at 72°C for 1 hr.

QMSP for 13 genes was performed using the 7500 Real-Time PCR System(Life Technologies, Carlsbad, CA). PCR was performed in a total volume of 20 μl, which consisted of 40 ng bisulfate-modified genomic DNA, 10 μl TaqMan Universal PCR Master Mix(Life Technologies, 0.25 μM of each primer, and 0.2 μM of the TaqMan probe. The primer and probe sequences that were used here are given in Additional file 1, Table S1. After an initial denaturation at 50°C for 2 min and 95°C for 5 min, 50 cycles at 95°C for 15 sec and 60°C for 1 min were performed.

The assays were repeated twice so as to confirm reproducibility, and the average was used for the subsequent data analysis. Data analysis was performed as previously described [28]. The values of QMSP that were obtained from each sample were first normalized using the value of *β*-*actin *as an internal reference. The values of QMSP that were obtained from the positive control were also normalized using the value of *β*-*actin*. The percent of methylated reference (PMR) was calculated as 100 × (normalized value of the sample)/(normalized value of the positive control). For statistical analysis, the PMR values that were less than 0.01 were rounded up to 0.01. Subsequently, the PMR values were converted to logarithms for statistical analysis.

## Results

### Somatic mutation analysis

The samples from patients 1-9 included pairs of primary and recurrent tumors, whereas those from patients 10-15 included multiple primary tumors. Except for patient no. 11, all HCC patients had backgrounds of viral infection. All of the clinical information is presented in Table 1.

**Table 1:** Summary of clinical information and experimental results.

					patient	HCC_ID	Clin. Diag.	HBsAg	HCV-Ab	p53	beta-catenin	GMA	common aberration	r
					1	2	MC	-	+			2.45	+	0.87
						4								
					2	6	MC	+	-	R273H	S33C	0	-	0.63
						8								
					3	10	MC	-	+			2.24	-	0.22
						12				Q129stop				
					4	14	MC	-	+	R249S		8.49	+	0.7
						16				S183stop, E298stop				
					5	18	MC	-	+		S33C	3.16	-	0.8
						21								
					6	102	MC	-	+	Q245C		4.9	-	0.83
						104				R249S	Q34E			
					7	106	IM	-	+			0	-	0.82
						108								
					8	110	IM	-	+			8.94	+	0.82
						112								
					9	114	MC	+	-			14.8	+	0.21
						116								
					10	26*	MC	-	+			0	-	0.27
						27*								
					11	29*	IM	-	-		Q34E	2	-	0.9
						30*					V22G,S33S,I35S			
					12	32*	IM	+	-	R175H		0	-	0.87
						33*								
					13	35*	MC	-	+	Y163N	D32G	10	+	0.63
						36*					T41I			
					14	38*	MC	-	+	F278A		14.3	+	0.4
						39*								
					15	41*	MC	+	-			1.41	-	0.76
						42*								

*, tumors dissected at the same operation. Others were sampled at different operations.

GMA, geometric mean of number of chromosomal aberrations; common aberrations, common chromosomal aberrations found in tumors from same patients; r, correlation coefficient of methylation patterns of tumors from same patients.

Genes subjected to the somatic mutation analysis - *TP53, CTNNB1 (β-catenin), CDKN2A*, and *KRAS *- were chosen using COSMIC (Catalog of Somatic Mutation in Cancer: http://www.sanger.ac.uk/genetics/CGP/cosmic/). The analysis was conducted using high-resolution melting and direct sequencing of the PCR products because direct sequencing often misses mutations on rare alleles; however, there was no discrepancy of results between two techniques. In our samples, no mutations were detected in *CDKN2A *or *KRAS*. As shown in Table 1, somatic mutations in *TP53 *and *CTNNB1 *were found to be associated with several tumors; however, tumors from the same patient did not share the same mutations. Furthermore, no mutations were found in the non-tumor counterparts (data not shown).

### Chromosomal aberration analysis

Chromosomal aberrations (gains and losses) were analyzed using the 44K human genome array, and the results are given in Table 2. Chromosome gains and losses at a probe-level resolution are given in Additional file 2, Table S2. The overviews of the aberrations were similar to those that have been published in previous studies [10,12,14]: frequent gains with 1q (9/30 in Table 2), 8q (12/30), and 20q (5/30) and losses with 1p (5/30), 4q (9/30), 8p (9/30), 13q (6/30), 16q (7/30), and 17p (10/30). Chromosomal aberrations that are common to multiple tumors are an important signature for tracing their histories, in that they indicate that these multiple tumors share a common lineage. Because array-CGH is sufficiently refined to determine breakpoints, we excluded aberrations that had different breakpoints at both ends from common aberrations. Tumors from six patients had common aberrations (Table 2, common aberration). For example, patient no. 13 had common aberrations in 1p and 17q: tumors no. 35 and 36 were estimated to have derived from an ancestor that had these aberrations.

**Table 2:** Results of array-CGH.

patient	HCC_ID	number of aberrations			geometric mean	common aberration	chrosome gain	chrosome loss
1	2	2			2.45	+	6p,8q	
	4	3					6p, 7q, 8q	
2	6	7			0	-		4q,8p,9p,9q,11q,16q,21q
	8	0						
3	10	5			2.24	-	4p,4q,17p,17q,22q	
	12	1					9q	
4	14	12			8.49	+	1q,11q	3pq,4q,8p,9pq,10q,11p,13pq,16q,17p,Yp
	16	6					14q,17q,19q	4q,11pq,13q
5	18	2			3.16	-	8q,Xq	
	21	5					7p	8p,12p,13q,17p
6	102	1			4.9	-	19p	
	104	24					1q,5p,5q,6p,8p,8q,11p,11q,20p,20q,Yp,Yq	1p,4p,4q,12p,12q,14q,15q,17p,18p,18q,21q,22q
7	106	0			0	-		
	108	18					5p,5q,6p,8q,13q,19p,19q,20p,20q,21q,22q,Xp,Xq,Yp	4q,8p,9p,13q
8	110	8			8.94	+	1q,8q,11pq,Yp,Yq	8p,16q,17p
	112	10					20q,Yp	1p,6q,8p,16p,16q,18q,Xp,Xq
9	114	11			14.8	+	2q,11q,22q	1p,1q,6q,8pq,10q,13q,16q,17p
	116	20					1q,6pq,7p,8q,13q,19q	1p,4q,6q,8p,9p,10p,12p,14q, 16p, 16q,17p,18q,19p,21q
10	26*	0			0	-		
	27*	0						
11	29*	1			2	-	8q	
	30*	4					1q,8q,Xq	10q
12	32*	1			0	-	20q	
	33*	0						
13	35*	25			10	+	1q,3p,3q,5p,5q,6p,6q,8p,8q,10p,10q,11p,11q 17q,18p,18q,19q,20p,20q,21q,Xp,Xq	4q,17p,Ypq
	36*	4					1q,17q	6q,17p
14	38*	12			14.3	+	1q,7pq,19q	1p,4q,5q,7q,8p,16q,17p,18p,18q
	39*	17					1q,3q,4p,8q,10q,19q,Xp,Xq	4q,7p,8p,9p,12p,13q,14q,17p,18q
15	41*	1			1.41	-	8q	
	42*	0						

*, tumors dissected at the same operation. Others were sampled at different operations. Common chromosomal aberrations are underlined.

**Figure 1:**
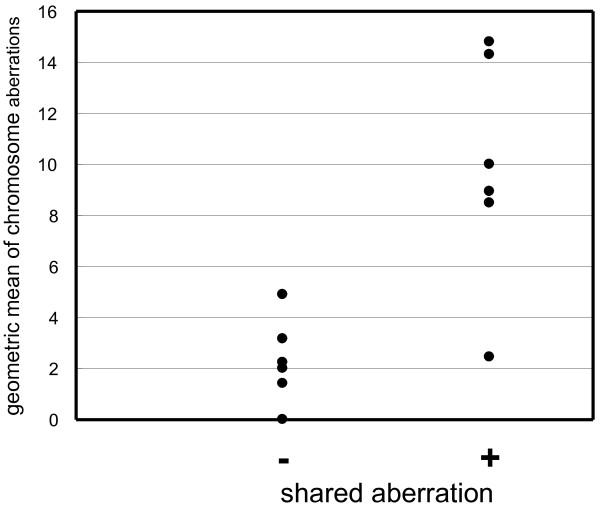
**The relationship between the occurrence of chromosomal aberrations that are common to tumors from the same patient and the average number of aberrations**. Dots represent cases. (+), Cases with common aberrations; (-), cases without common aberrations.

As shown in Table 2, the number of chromosomal aberrations varied as a function of tumor in different patients as well as in the same patient. Figure 1 depicts the geometric mean aberration number in each patient, grouped according to presence or absence of common aberrations. There is a marked difference in the numbers of aberrations between the two groups (Mann-Whitney test, *p *< 0.01). Thus, the identification of common aberrations depends on the total number of chromosomal aberrations.

### Methylation analysis

**Figure 2:**
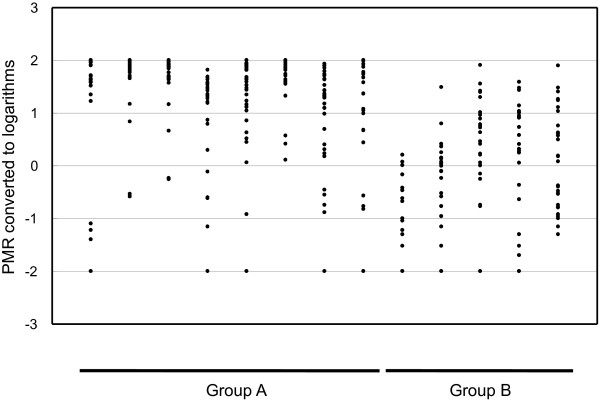
**The promoter methylation of genes.** Vertical axis, PMR values that are converted to common logarithms; horizontal axis, genes that are subjected to promoter methylation analysis.

The genes that were subjected to the analysis of promoter methylation were mostly tumor suppressor genes, which are methylation markers that are widely used in cancer studies. The gene set included all of the genes that have been used in recent related studies [16,23]. We performed QMSP using all of the tumor samples as well as adjacent non-tumor samples. The results (PMR) are given in Additional file 3, Table S3. In most cases, the amplification using non-tumor samples was less or far less than the detection level using HCC (Additional file 3, Table S3), confirming the cancer-specific methylation of the genes. In HCC, the degree of methylation differed as a function of the type of gene. Group A genes tended to have high PMR values, whereas group B genes tended to have diverse PMR values (Figure 2). QMSP has a wide dynamic range and is sufficiently sensitive to detect a methylation as low as 0.02%.

**Figure 3:**
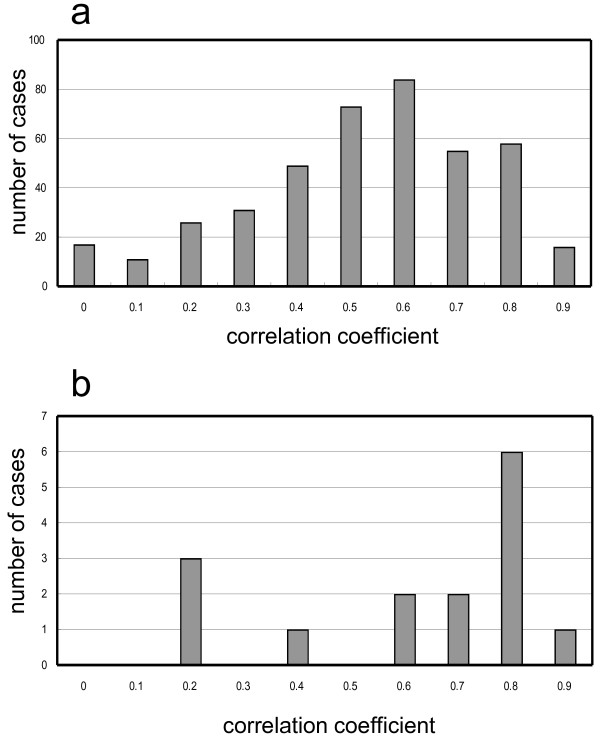
**A histogram of correlation coefficients of methylation patterns**. Vertical axis, number of cases; horizontal axis, correlation coefficient ranges. For example, 0.8 represents values that range from 0.8 to 0.9. "0" includes cases from -0.14 to 0. a) All possible combinations of tumors from different patients; b) tumors from the same patient.

Because the methylation status that is described by the PMR value is not discrete, we compared the overall patterns by calculating the correlation coefficient (*r*). First, we calculated the r of log-converted PMRs using all of the possible combinations of tumors from different patients. The distribution of r is shown in Figure 3a. The distribution ranged from 0.96 to -0.14, and the median was 0.601. This is a control distribution that was obtained from sample pairs of completely different origins. The distribution of r of tumors from the same patient is shown in Figure 3b - this distribution is shifted to the right, suggesting a greater similarity in methylation patterns in tumors from the same patient. In total, 47% (7/15) of cases had an r that was greater than 0.8 (Figure 3b). In contrast, only 18% of tumors from different patients had an *r *that was greater than 0.8 (Figure 3a). The chance of more than seven cases for which *r *was more than 0.8 out of 15 sample pairs from different patients was deduced by randomly sampling 15 cases from the pool of pairs from different patients: 110 successes per 100,000 trials. The high similarity in methylation patterns in the same patient was, thus, statistically significant. It should be noted that all clinical IM cases had values of *r *that were greater than 0.8 (*p *= 0.025, Fischer's exact test) (Table 1). Despite the detailed examination of the methylation in individual genes, we could not find any rule for the high similarity. The high similarity of the overall methylation patterns is a potential indicator of IM.

## Discussion

The origin of individual tumors in HCC is a major issue; that is, whether they are derived from a common precancerous or cancerous ancestor or individually from hepatocytes. We can deduce how individual tumors evolved from common ancestor cells by comparing the aberration patterns of multiple genetic aberrations. This could lead to the differential diagnosis of IM and MC, which is often important when making a therapeutic decision. The initial step of the analysis is to find molecular genetic features that are common to individual tumors. Many efforts have been made to determine common genetic changes [17-23] and to correlate them with a clinical diagnosis; however, as described earlier, there is still no consensus. In this report, we evaluated somatic mutations, chromosomal aberrations, and promoter methylation. The latest techniques, such as array-CGH and QMSP, were used for the first time for the analysis of multiple HCC in this study.

The occurrence of somatic mutations was too rare to identify common aberrations. Despite the initial effort to use p53 for the differential diagnosis [17], we found that it is mutated in only a few cases, which was similar to previous reports [29,30]. We found common chromosomal aberrations in six cases, wherein multiple tumors were likely to have derived from a common lineage; however, the occurrence of chromosomal aberrations differed within tumors, as well as among tumors from the same patient. In cases with infrequent chromosomal aberrations, it was difficult to deduce the history of tumors from the aberration pattern. Even tumors with common aberrations possessed different aberrations and were not clones. In contrast, we found, from quantitative analysis of CGH patterns, that there was no substantial heterogeneity in each tumor (data not shown). These observations suggest that biological IM tumors, that is, clonal tumors, were rare in comparison to biological MC tumors, that is, multiple tumors with different genotypes, which strongly contrasts with recent observations in other cancers that have been obtained by large-scale sequencing. For example, recent work in colorectal cancer has demonstrated that more than 90% of somatic mutations were simultaneously present in different malignant tumors; that is, a primary tumor versus its metastases or a primary tumor versus a recurrent tumor in the same patient [31].

As previously described, the choice of therapy often depends on the clinical diagnosis of IM and MC [5,6]. Multiple HCC is diagnosed as IM when the primary tumor is moderately or poorly differentiated, and multiple tumors appear within two years after surgery. The diagnosis of MC is achieved when multiple tumors are highly differentiated and appear with hepatitis or cirrhosis; however, these criteria have no direct correlation with the process of carcinogenesis. Therefore, an exploration of the molecular genetic differences between IM and MC does not have a solid scientific basis. Thus, it is not surprising that there has been no consensus in the molecular diagnostic criteria for IM and MC. Our data concerning somatic mutations and chromosomal aberrations suggest that biological IM is likely to be rare. Difficulty in the molecular differentiation of IM and MC is at least partly due to the rarity of biological IM.

All IM cases exhibited a similar methylation pattern. Unlike genetic changes, epigenetic changes were not necessarily irreversible. Here, a similar methylation pattern for multiple HCC would reflect the environmental factors that surrounded their development rather than their derivation from a common ancestor because the data concerning somatic mutations and chromosomal aberrations suggest the rarity of biological IM. Although confirmation with a larger number of patients is still required, the methylation pattern may be useful in the clinical diagnosis of marginal cases.

In general, current techniques do not offer adequate information on the carcinogenesis of multiple HCC. There is also a possibility that the negative results are due to the small sample size. Recently, sequencers based on a new principle have appeared, and the rate of sequence data production has improved by more than 100 times and is still increasing [32]. The lineage of multiple tumors and liver tissues and the process of carcinogenesis will be identified when the somatic mutations are revealed by the entire genomic sequencing of multiple HCC.

## Conclusions

The overall scarcity of common somatic mutations and chromosomal aberrations suggest that biological IM is likely to be rare. Tumors from the same patient had a methylation pattern that was more similar than tumors from different patients. Because all clinical IM cases were highly similar, methylation patterning may be applicable to support the clinical diagnosis of IM and MC.

## Competing interests

The authors declare that they have no competing interests.

## Authors' contributions

KT designed and carried out all of the molecular genetic studies. TY and YS collected the tumor and normal tissues and are responsible for the clinical components of the study. KK designed the study, participated in its design and coordination, and wrote the manuscript. All authors read and approved the final manuscript.

## Pre-publication history

The pre-publication history for this paper can be accessed here:

http://www.biomedcentral.com/1471-2407/10/530/prepub

## Supplementary Material

Additional file 1 **Table S1**. Click here for file

Additional file 2 **Table S2**. Click here for file

Additional file 3 **Table S3**. Click here for file
